# The Antioxidant Capacity and Flavor Diversity of Strawberry Wine Are Improved Through Fermentation with the Indigenous Non-*Saccharomyces* Yeasts *Hanseniaspora uvarum* and *Kurtzmaniella quercitrusa*

**DOI:** 10.3390/foods14050886

**Published:** 2025-03-05

**Authors:** Ruipeng Wang, Bo Yang, Saihong Jia, Yiwei Dai, Xinping Lin, Chaofan Ji, Yingxi Chen

**Affiliations:** SKL of Marine Food Processing & Safety Control, National Engineering Research Center of Seafood, Collaborative Innovation Center of Seafood Deep Processing, School of Food Science and Technology, Dalian Polytechnic University, Dalian 116034, China; xxl1917060202@163.com (R.W.); 18242039327@163.com (B.Y.); 15131067068@163.com (S.J.); ywdai6228@126.com (Y.D.); yingchaer@163.com (X.L.); jichaofan@outlook.com (C.J.)

**Keywords:** strawberry wine, non-*Saccharomyces* yeast, fermentation condition, antioxidant capacity, flavor diversity

## Abstract

The production of strawberry wine is an effective strategy for addressing the significant economic losses caused by strawberry spoilage. In recent years, there has been an increase in consumer demand for quality and flavor diversity in fruit wines. Therefore, it is necessary to develop novel strawberry wine products. In this research, we assessed and analyzed the influences of fermentation with *Hanseniaspora uvarum*, *Kurtzmaniella quercitrusa*, and *Saccharomyces cerevisiae* under four fermentation conditions on the fermentation kinetics, organoleptic characteristics, chemical compositions, antioxidant capacities, and flavor profiles of strawberry wines. Strawberry wines fermented with the indigenous non-*Saccharomyces* yeasts *H. uvarum* and *K. quercitrusa* showed higher 2,2-Diphenyl-1-picrylhydrazyl (DPPH) and 2,2′-Azinobis-(3-ethylbenzothiazoline-6-sulfonate) (ABTS) free-radical-scavenging capacities and significantly different flavor profiles compared to strawberry wines fermented with *S. cerevisiae*. In addition, adjusting the initial soluble solids contents of strawberry juices and fermentation temperatures positively affected the quality and flavor profiles of strawberry wines fermented with the *H. uvarum* and *K. quercitrusa* strains. Under the condition of 18 °C–20 °Brix, strawberry wine fermented with *K. quercitrusa* presented the highest antioxidant capacity, with enhanced flavor diversity and color intensity. It is worth noting that *K. quercitrusa* can be an alternative yeast for producing high-quality strawberry wine with a distinct floral aroma.

## 1. Introduction

The garden strawberry (*Fragaria* × *ananassa*) is one of the world’s most popular fruits due to its tasty flavor and richness in nutrients. Moreover, it is also rich in substances with antioxidative capacity which have been demonstrated to have anti-inflammatory and free-radical-scavenging capabilities [1,2]. In China, strawberries are recognized as a pivotal economic fruit crop, and it has the most extensive acreages and the highest annual yield in the world [3]. There is a bumper crop for strawberries every spring, but strawberries are an extremely perishable fruit, resulting in unnecessary waste and serious economic losses. Processing fresh strawberries that cannot be sold in a timely manner into high-value products is a favorable approach that can help to improve the economic returns of both food enterprises and fruit growers.

Strawberry wine is known as a valuable strawberry by-product, with an attractive ruby red color and an intense aroma, and it preserves the antioxidant capacity and nutrients of strawberries [3]. However, in recent years, consumers have been demanding higher quality and more personalized fruit wines, including fruit wines with a low alcohol content, high antioxidant capacity, or unique flavor. Many studies have revealed that the involvement of non-*Saccharomyces* yeasts in the fermentation process can improve the quality and flavor diversity of fruit wines while also providing an alternative strategy for the fermentation of low-alcohol fruit wines [4,5,6]. Current studies on non-*Saccharomyces* yeasts have focused on wine and cider, while relevant research on strawberry wine is still limited. The non-*Saccharomyces* yeast strain *Torulaspora delbrueckii* was used to develop a fermented strawberry wine, and it significantly increased the anthocyanin content and improved the sensory characteristics and overall quality of the strawberry wine compared to *Saccharomyces cerevisiae* [7]. However, the effects of other non-*Saccharomyces* yeasts species on the physicochemical parameters, chemical compositions, antioxidant capacities, and organoleptic characteristics of fermented strawberry wines are still unknown.

Fruit wine fermentation is a complex process in which both the fermentation temperature and the initial soluble solids (SSC) content of the juice have different effects on the performance of non-*Saccharomyces* yeast. Fermentation by non-*Saccharomyces* yeasts at low temperatures facilitated the retention of polyphenolic compounds, maintained the stability of anthocyanins, have been shown to reduce the degradation of vitamin C and produce more organic acids and esters, effectively improving the antioxidant capacity and flavor profile of the wine [8]. Differences in the initial soluble solids contents of fruit juices affect the growth and metabolism of non-*Saccharomyces* yeasts during fermentation, which, in turn, influences the production of volatile organic compounds such as alcohols and esters, resulting in an altered flavor profile and diversity in fruit wines. Fermentation with non-*Saccharomyces* yeast and apple juice with a high SSC resulted in a significant increase in the esters content of the fermented cider, particularly the ethyl acetate content and ethyl isovalerate content, which resulted in more fruity and floral flavours in the cider [9]. Therefore, investigating the effects of fermentation temperatures and the SSCs of strawberry juices on strawberry wines fermented with different non-*Saccharomyces* yeasts is an effective approach for developing strawberry wines with high quality and flavor diversity.

The effects of *Hanseniaspora uvarum* or *Kurtzmaniella quercitrusa* as single fermentation starters on the quality and flavor profiles of fermented strawberry wines was unknown. In a previous study, we obtained indigenous *H. uvarum* and *K. quercitrusa,* with favorable performances in fruit wine fermentation, by isolation and screening from fruits. In addition, the effects of fermentation temperatures and the SSCs of strawberry juices on the fermentation characteristics of non-*Saccharomyces* yeasts have been less frequently investigated. Therefore, the major objective of this research was to assess and comparatively analyze the organoleptic characteristics, chemical compositions, antioxidant capacities, and flavor profiles of strawberry wines produced by an indigenous *H. uvarum* strain, an indigenous *K. quercitrusa* strain, and a *S. cerevisiae* strain, respectively, under four fermentation conditions. Our findings not only highlight the functions of the indigenous *H. uvarum* and *K. quercitrusa* strains in enhancing the antioxidant capacities and flavor diversity of strawberry wines but also provide a reference for developing high-quality strawberry wine products.

## 2. Materials and Methods

### 2.1. Materials

Frozen strawberries were purchased from a strawberry orchard in Dandong (Liaoning Province, China) and frozen at −20 °C until processing. The control group *Saccharomyces cerevisiae* strain, CGMCC 2.3854 (Sc), was purchased from the China General Microbiological Culture Collection Center. The indigenous *Hanseniaspora uvarum* (Hu) and *Kurtzmaniella quercitrusa* (Kq) strains with favorable performances in fruit wine fermentation were screened and isolated from fruits. The media components, reagents, and chemicals were obtained from Aladdin (Shanghai, China), Damao (Tianjin, China), Shyuanye (Shanghai, China), and Macklin (Shanghai, China).

### 2.2. Strawberry Wine Fermentation

The frozen strawberries were processed into strawberry juice by thawing, juicing, enzymatic hydrolysis, and filtration [10]. Then, glucose was added to adjust the soluble solid content (SSC) of the strawberry juice to 11 °Brix or 20 °Brix, and the SO_2_ concentration was increased to 60 mg/L with K_2_O_5_S_2_ to avoid contamination. For each fermentation sample, 100 mL of adjusted strawberry juice was added into a 250 mL Duran bottle and then pasteurized (97 °C, 1 min). The Hu, Kq, and Sc strains were activated in liquid cultures before inoculation, and then they were individually inoculated into the pasteurized strawberry juices at 10^6^ CFU/mL. In this investigation, four different fermentation conditions were set, as follows: 25 °C–11 °Brix, 25 °C–20 °Brix, 18 °C–11 °Brix, and 18 °C–20 °Brix [11,12,13,14]. Fermentation was considered complete when the SSCs of the strawberry wines did not change significantly for two consecutive days. The strawberry wine samples were centrifuged at 8000× *g* for 10 min to remove precipitates, and they were preserved at −20 °C.

### 2.3. Basic Parameters Analysis of the Strawberry Wine

The ethanol and glucose contents were determined as previously described via the enzymatic autoanalyzer SBA-90 (Biology Institute of Shandong Academy of Sciences, Jinan, China) [15]. The SSC was evaluated with a handheld refractometer (HSU-32, Shanghai Precision Instrument Co., Ltd., Shanghai, China), and the CO_2_ lost weight was quantified using an electronic scale (Sartorius Scientific Instrument Co., Ltd., Beijing, China) [16]. The content of titratable acid was measured by the acid-base titration method and expressed as the concentration of citric acid [17]. We diluted 1 mL of the sample to 50 mL, and 200 μL of a 1% phenolphthalein–ethanol solution was added. The solution was then titrated with 0.1 mol/L NaOH until it turned slightly red. If the red color did not fade within 30 s, it was considered as the end point of the titration, and the volume of 0.1 mol/L of NaOH was used to calculate the content of titratable acid. The content of reducing sugar was measured by the 3,5-dinitrosalicylic acid (DNS) method [18]. The pH, glycerol concentration, color intensity (CI), and color tonality (CT) of the strawberry wines were determined as previously described [10,19].

### 2.4. Determination of Active Substances Content and Antioxidant Capacity

The total phenol content (TPC) was determined by the Folin–Ciocalteu reagent method [20]. The total flavonoid content (TFC) was measured by the aluminum chloride colorimetric method as described previously by Wu et al. [21], with minor modifications. The strawberry wine samples (1 mL) were mixed with 0.2 mL of NaNO_2_ (5% *w*/*v*) and allowed to stand for 6 min, then 0.2 mL of AlCl_3_ (10% *w*/*v*) was added and allowed to react for 6 min. Finally, 2 mL of NaOH (4% *w*/*v*) was added and diluted to a final volume of 5 mL with ddH_2_O, then it was left for 15 min before analysis. The TFC was analyzed by measuring the 510 nm absorbance of the mixed liquor, and the TFC was expressed as the rutin equivalent (mg/L). The determination of the total anthocyanin content (TAC) was carried out according to the procedure prescribed by the Association of Official Analytical Chemists [22].

The 2,2-Diphenyl-1-picrylhydrazyl (DPPH) and 2,2′-Azinobis-(3-ethylbenzothiazoline-6-sulfonate) (ABTS) free-radical-scavenging capacities of the strawberry wines were determined to evaluate their in vitro antioxidant capacities [23]. For the DPPH assay, 0.2 mmol/L of DPPH solution was prepared with anhydrous ethanol. We missed 1 mL of a 200-fold dilution of strawberry wine with 1 mL of the DPPH solution, and then the mixture was incubated in the dark at 25 °C for 30 min and the absorbance of the mixture was measured at 517 nm. We used 0, 1, 2, 3, 4, 5, 6, and 7 mg/L of gallic acid to determine the standard curves under the same conditions. The DPPH free-radical-scavenging capacities of the samples were calculated through the standard curve and expressed as the gallic acid equivalents (mg/L). For the ABTS assay, 100 mL of 14 mM ABTS and 100 mL of 5 mM potassium were mixed and allowed to react at 25 °C for 12 h. The absorbance of the mixture was adjusted to OD_734nm_ = 0.7 using an 80% ethanol solution to obtain the ABTS solution. Then, 100 μL of a 30-fold dilution of the sample was mixed with 900 μL of the ABTS solution in a dark environment for 15 min at 25 °C, and the absorbance of the mixture was measured at 734 nm. We used 0, 20, 40, 60, 80, 100, 120, and 140 mg/L of Trolox to determine the standard curves under the same conditions. The ABTS free-radical-scavenging capacities of the samples were calculated through the standard curve and expressed as the Trolox equivalents (mg/L).

### 2.5. Profiling of the Volatile Organic Compounds

The volatile organic compounds (VOCs) in the strawberry wines were identified and determined by a GC-IMS (FlavourSpec, G.A.S., Dortmund, Germany) equipped with an MXT-WAX Cap. Column (30 m × 0.53 mm × 1 μm) using a previously described method with a slight modification [24]. A 1 mL sample and 10 μL of 2-octanol (600 mg/L) were mixed and incubated in a headspace vial at 60 °C for 10 min. The VOCs were identified by comparing the gas chromatographic retention indices and drift times with standards from the GC-IMS database (G.A.S., Dortmund, Germany).

The GC-MS (7890A-5975C, Agilent, Palo Alto, CA, USA) equipped with an HP-5MS (30 m × 0.25 mm × 0.25 μm) was also utilized to determine and analyze the VOCs in the samples [25]. A sample (2 mL) was added into a 20 mL headspace vial, then 2-octanol (internal standard, 600 mg/L, 20 μL) was added to facilitate a semi-quantitative analysis. The VOCs were identified by comparing the data with the NIST 14 mass spectral library. The semi-quantitation analysis was applied to quantify the relative content of the VOCs.

### 2.6. Electronic Nose Analysis

The strawberry wines were analyzed using a PEN3 electronic nose (Airsense Analytics, Inc., Schwerin, Germany) according to the method used by Liu et al. [26]. The electronic nose was equipped with 10 metal oxide semi-conductor sensors, which were used to characterize the VOC patterns in the strawberry wines. Briefly, 2 mL of strawberry wine was added into a 20 mL airtight vial and incubated at 60 °C for 20 min. The headspace gas was pumped into the sensor cavity by using clean air as a carrier gas at a flow rate of 200 mL/min. Each measurement cycle lasted 100 s, with a 1 s interval for data collection. Between measurement cycles, the sensors were cleaned with clean air for 5 min.

### 2.7. Statistical Analysis

The GC-IMS data were analyzed using VOCal 0.1.0 software for fingerprint mapping. A principal component analysis (PCA) was performed and plotted using SIMCA 14.1 software. The line graphs, bar graphs, and radar plots were plotted with Origin 2021 software. The significance of difference tests were performed on the experimental data by SPSS 19 with a one-way analysis of variance and a Duncan test.

## 3. Results and Discussion

### 3.1. Fermentation Kinetics

**Figure 1:**
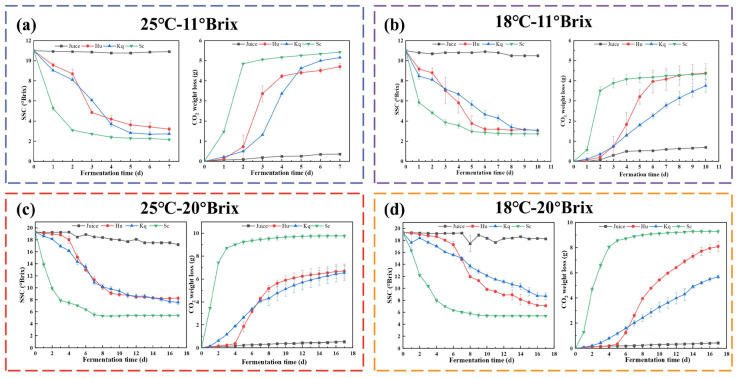
The variations in SSC and the CO_2_ weight loss during fermentation with the Hu, Kq, and Sc strains under the four fermentation conditions: (**a**) 25 °C–11 °Brix, (**b**) 18 °C–11 °Brix, (**c**) 25 °C–20 °Brix, and (**d**) 18 °C–20 °Brix. Juice, unfermented strawberry juice; Hu, pure inoculation of *H. uvarum*; Kq, pure inoculation of *K. quercitrusa*; Sc, pure inoculation of *S. cerevisiae*.

The variations in SSC and the CO_2_ weight loss during fermentation of the Hu, Kq, and Sc strawberry wines under four fermentation conditions are shown in Figure 1. Under the four fermentation conditions, the Sc strain showed the fastest fermentation rate, and it was able to complete fermentation at least 3 days earlier than the Hu and Kq strains. During strawberry wine fermentation, the Sc strain showed a better fermentation performance than the non-*Saccharomyces* yeasts Hu and Kq, in agreement with the previous research findings [27]. The 25 °C–11 °Brix condition showed the shortest fermentation cycles, and as the temperature decreased and the initial SSC increased, the fermentation rates were reduced and the fermentation cycles were lengthened [28]. Compared to the 25 °C–11 °Brix condition, under the 18 °C–20 °Brix condition, the fermentation cycle of the Sc group was lengthened from 4 days to 8 days, and the fermentation cycle of the Hu and Kq groups was lengthened from 7 days to 17 days.

### 3.2. Physicochemical Parameters

**Figure 2:**
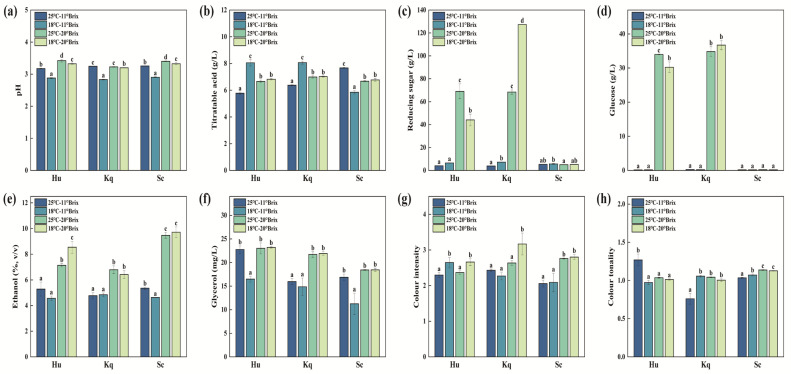
Physicochemical parameters of the strawberry wines with different fermentation trials: (**a**) pH value, (**b**) titratable acid content, (**c**) reducing sugar content, (**d**) glucose content, (**e**) ethanol content, (**f**) glycerol content, (**g**) color intensity, and (**h**) color tonality. The values with different superscript roman letters in the same figure are significantly different according to the Duncan test (*p* < 0.05).

Under the same initial SSC condition, the pH of the 25 °C strawberry wines showed markedly higher values compared to the 18 °C strawberry wines (Figure 2a), which indicated that fermentation at a low temperature facilitated the formation and accumulation of acids in the strawberry wine [29]. Previous research findings have demonstrated that low-temperature fermentation affects the metabolism of yeasts and leads to the formation of secondary metabolites, including acetic acid and succinic acid [30]. No significant differences were found in the glucose contents among the Sc strawberry wines under the four fermentation conditions. However, for the Hu and Kq strains, the reducing sugar contents (44.05–127.38 g/L) and glucose contents (30.25–36.71 g/L) of the 20 °Brix strawberry wines were significantly higher than those of the 11 °Brix strawberry wines (Figure 2c,d). These results demonstrated that the Hu and Kq strains had limited fermentation capacities, which was agreement with previous reports [6,31]. The differences in ethanol contents mainly depended on the initial SSC conditions, while the yeast species had a smaller effect. For all three strains, the ethanol contents of the 20 °Brix strawberry wines were significantly higher than those of the 11 °Brix strawberry wines. It is worth noting that under the initial 20 °Brix condition, the Kq strawberry wines had significantly lower ethanol contents than those of the Sc strawberry wines. (Figure 2e). This finding suggested that the non-*Saccharomyces* Kq strain could be an alternative yeast for producing low-alcohol fruit wine. As shown in Figure 2f, increasing the initial SSC resulted in an elevated glycerol content, which could improve the lubricity of the strawberry wine mouthfeel [32]. Comparing the four conditions, the 18 °C–20 °Brix condition showed the highest CI values, which indicated that the colors of the Hu, Kq, and Sc strawberry wines fermented under this condition were brighter and more intense [19], among which the Kq sample showed the highest CI value (3.17 ± 0.31) (Figure 2g).

### 3.3. Active Substances Content and Antioxidant Capacity

**Figure 3:**
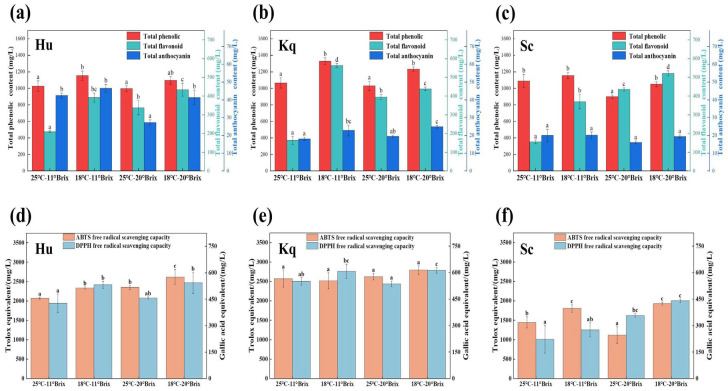
Total phenolic contents, total flavonoid contents, total anthocyanin contents, and in vitro antioxidant capacities of the different strawberry wine samples: (**a**) total phenolic contents, total flavonoid contents, and total anthocyanin contents of the Hu samples; (**b**) total phenolic contents, total flavonoid contents, and total anthocyanin contents of the Kq samples; (**c**) total phenolic contents, total flavonoid contents, and total anthocyanin contents of the Sc samples, (**d**) DPPH and ABTS free-radical-scavenging capacities of the Hu samples; (**e**) DPPH and ABTS free-radical-scavenging capacities of the Kq samples, and (**f**) DPPH and ABTS free-radical-scavenging capacities of the Sc samples. The values with different superscript roman letters in the same figure are significantly different according to the Duncan test (*p* < 0.05).

Phenolics, flavonoids, and anthocyanins contribute significantly to the appearance, antioxidant capacity, and organoleptic characteristics of fruit wine [33]. The fermentation temperature was the main factor influencing the TPC, TFC, and TAC in the strawberry wines. Compared to the 25 °C strawberry wines, the 18 °C strawberry wines showed a 0.16–0.25-fold increase in TPC, a 0.11–2.46-fold increase in TFC, and a 0.00–0.51-fold increase in TAC (Figure 3a–c). These results demonstrated that low-temperature fermentation had a positive effect on the retention or production of active substances in the strawberry wines, resulting in an enhancement of the quality of the strawberry wine. The ABTS and DPPH free-radical-scavenging capacities of the Hu and Kq strawberry wines were higher than those of the Sc strawberry wines under all fermentation conditions (Figure 3d–f), which was consistent with previous studies showing that non-*Saccharomyces* strains have the potential to increase antioxidant capacity in fruit wines [34,35]. Moreover, the fermentation of fruit wines at low temperatures has previously been reported to result in increased antioxidant capacity [36,37]. Consistently, under the same initial SSC condition, the ABTS and DPPH of the 18 °C strawberry wines were higher than those of the 25 °C strawberry wines. In addition, under the 18 °C–20 °Brix condition, the Kq strawberry wine had the highest ABTS (2793.12 ± 111.97 mg/L Trolox equivalent) and DPPH (612.60 ± 16.17 mg/L GAE equivalents) free-radical-scavenging capacities. These findings highlight not only that the combination of the non-*Saccharomyces* yeast and low temperature fermentation conditions could be a viable strategy for improving the antioxidant capacity of fruit wine but also revealed that the non-*Saccharomyces* Kq strain has the potential to produce fruit wine with a high antioxidant capacity.

### 3.4. Volatile Organic Compounds

#### 3.4.1. GC-IMS Analysis

**Figure 4:**
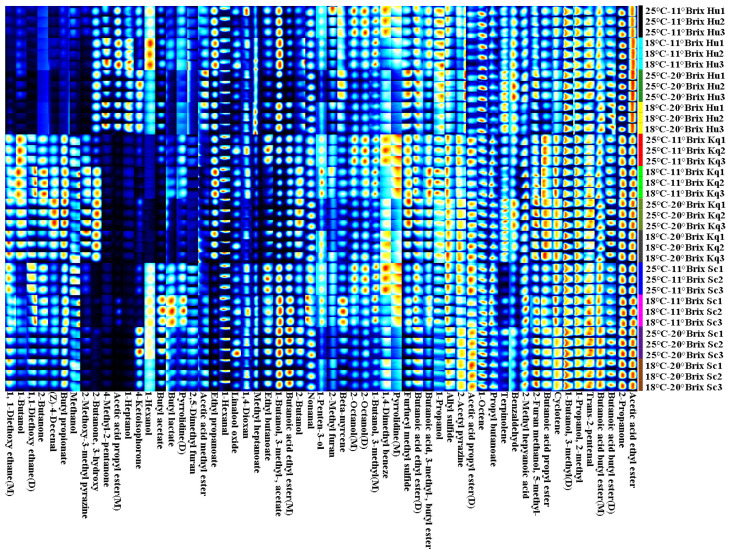
GC-IMS fingerprints of the volatile organic compounds in the strawberry wines fermented in the different fermentation trials. M, protonated monomer; D, proton-bound dimer.

Flavor diversity in fruit wine is one of the most important factors in determining consumer purchase intention, and it is primarily influenced by the types and contents of volatile organic compounds (VOCs) in fruit wines [38]. The fingerprint demonstrated the characteristic distribution of C3-C10 VOCs in the strawberry wine samples (Figure 4). A total of 52 VOCs were identified, including 15 esters, 12 alcohols, 4 aldehydes, 5 ketones, 1 acid, and 15 others. The strawberry wines fermented under the different conditions showed variability in the flavor profiles, mainly due to the differences in inoculation yeast strains and initial SSCs. As shown on the left side of Figure 4, there was a clear difference in the VOCs between the Sc, Kq, and Hu strawberry wines, indicating that yeast strains could alter flavor diversity in strawberry wines. The Hu strawberry wines contained higher contents of 4-methyl-2-pentanone, 1-heptanol, 4-ketoisophorone, and 1-hexanol, endowing the wine with more woody, sweet tea, fruity, and floral notes [39]. The contents of 1-butanol, butyl propionate, 2-methoxy-3-methyl pyrazine, and acetoin (2-butanone, 3-hydroxy) were the highest in the Kq strawberry wines, which brought the pungent, apple, grilled nut, and creamy notes to the Kq samples [40]. The contents of 1-hexanal, 1-penten-3-ol, butyl isovalerate (butanoic acid, 3-methyl-butyl ester), butyl acetate, and 1-propanol in the 11 °Brix strawberry wines were higher than those in the 20 °Brix strawberry wines. It is possible that increasing the initial SSC prolongs the fermentation cycle of strawberry wine, promoting further metabolic conversion of these VOCs by the yeast cells. Among them, 1-penten-3-ol is a key compound used to identify strawberry ripeness, with a mild grassy note [41]. Butyl isovalerate is one of the characteristic flavor compounds of strawberries, produced mainly by the metabolism of fatty acids and amino acids in the late ripening stage, creating apple and banana notes [42,43]. The gradual increase in the content of butyl acetate (pineapple and banana notes) as a strawberry grows has been previously described [44,45]. Moreover, the 20 °Brix strawberry wines showed higher contents of terpinolene, butanoic acid butyl ester, isoamyl alcohol (1-butanol, 3-methyl), and acetic acid ethyl ester, which brought pine, tropical fruit, and rose notes to the wines [46,47]. Isoamyl alcohol, one of the major metabolites of yeast cells, is synthesized via the Harris pathway and the Ehrlich pathway, which could enhance the flavor profile of strawberry wines [48].

#### 3.4.2. GC-MS Analysis

**Figure 5:**
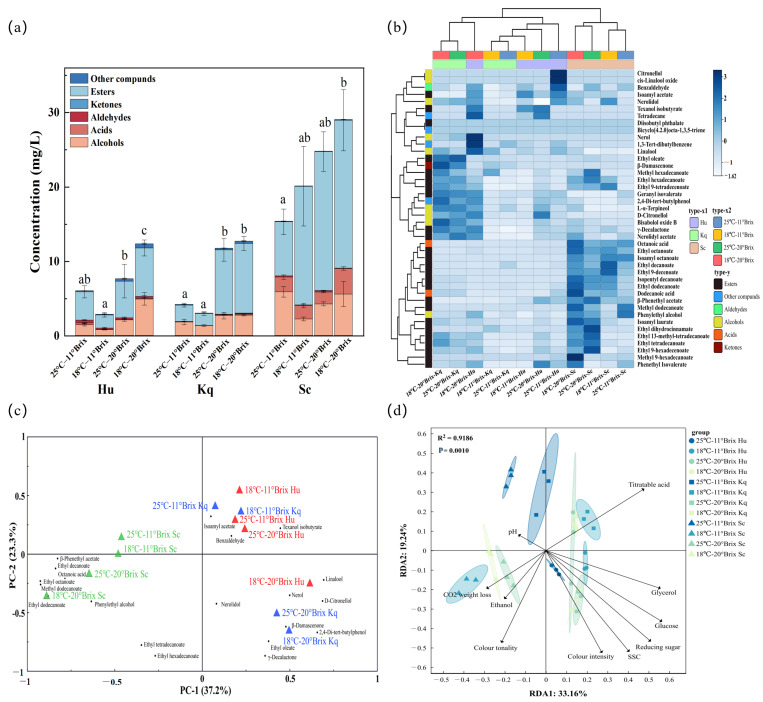
Overview of the overall C7–C22 volatile organic compounds in the different strawberry wine samples: (**a**) overview of the volatile organic compound content profiles. Note: the lowercase letters (a, b, c, etc.) in the figure indicate the significance of different fermentation conditions, (**b**) hierarchical clustering and heat map visualization of the volatile organic compounds in the different samples, (**c**) the principal components analysis (PCA) biplot of the volatile organic compounds with a relative odor capacity value of greater than 1 (rOAV ≥ 1) in the different strawberry wines, and (**d**) a redundancy analysis (RDA) of the volatile organic components and physicochemical parameters of the different strawberry wines.

The C7-C22 VOCs in strawberry wines were qualitatively and semi-quantitatively analyzed by GC-MS. A total of 42 VOCs were detected, including 25 esters, 9 alcohols, 2 acids, 1 aldehyde, 1 ketone, and 4 other compounds (Table S1). The total VOC contents of the strawberry wines were mainly influenced by the fermentation strains and the initial SSC (Figure 5a). For all three yeast strains, the 20 °Brix strawberry wines had significantly higher total VOC contents than those of the 11 °Brix strawberry wines. An increase in the initial SSC content promoted the growth and metabolism of the yeast strains, which contributed to facilitating the production of specific VOCs, leading to increases in the flavor intensities of the strawberry wines [49]. The initial SSC had the most significant impact on the total VOC contents in the Kq strawberry wines, and compared to the 11 °Brix Kq strawberry wines, the 20 °Brix Kq strawberry wines showed 1.78–3.19-fold increases in the total VOC contents and 2.91–4.92-fold increases in the ester compound contents. Under all four different fermentation conditions, the total VOC contents of the Sc strawberry wines were higher than those of the Hu and Kq strawberry wines. In addition, under the 18 °C–20 °Brix condition, all strawberry wines had their highest total VOC contents, among which the Sc strawberry wine had the highest total VOC content (29.06 ± 5.81 mg/L). Therefore, the 18 °C–20 °Brix condition was the optimal fermentation condition to accumulate the total VOC contents of the strawberry wines. Figure 5b shows the differences in the flavor profiles of the Sc, Hu, and Kq strawberry wines fermented under the four fermentation conditions. Comparing the strawberry wines fermented by the different strains, the relative abundances of ethyl esters, volatile acids, and higher alcohols were higher in the Sc strawberry wines. In comparison, the Hu and Kq strawberry wines contained higher relative abundances of terpene alcohols and ketones. These results demonstrated that the Hu and Kq strains could efficiently alter the flavor profile of strawberry wine and that a mixed fermentation of non-*Saccharomyces* yeasts with the *S. cerevisiae* strain could be an effective strategy for simultaneously obtaining high VOC contents and increasing flavor diversity in fruit wines [50].

#### 3.4.3. Principal Component Analysis

The odor thresholds of VOCs vary considerably; therefore, it is necessary to take odor thresholds into account when determining the impact of VOCs on the aroma characteristics of a fruit wine. The relative odor capacity value (rOAV), the ratio of VOC content to odor threshold, can be utilized to quantitatively assess the extent to which VOCs contribute to the overall flavor. It is generally accepted that VOCs with an rOAV of ≥1 can be identified as key flavor compounds and have a remarkable effect on the flavor of fruit wines [51,52,53]. A principal component analysis (PCA) was performed on 20 VOCs with rOAV values of ≥1 (Table S2) and 12 fermentation groups in order to elucidate the key aroma characteristics of the different samples (Figure 5c). Principal component 1 (PC1) accounted for 37.2% of the total variance and effectively discriminated between the strawberry wines with different fermentation strains, with the Sc strawberry wines on the negative axis of PC1 and the other strawberry wines on the positive axis of PC1. Principal component 2 (PC2) accounted for 23.3% of the total variance and was effective in differentiating the strawberry wines with different initial SSCs. All of the 11 °Brix strawberry wines and 25 °C–20 °Brix Hu strawberry wines were on the positive axis of PC2 with 3 key flavor compounds, while the remaining 20 °Brix strawberry wines were on the negative axis of PC2 with 17 key flavor compounds.

The 20 °Brix Sc strawberry wines were located in the third quadrant with β-phenethyl acetate, ethyl decanoate, octanoic acid, ethyl octanoate, methyl dodecanoate, ethyl dodecanoate, and phenylethyl alcohol, creating more pear, citrus, honey, rose, and green grass notes in the wines. Among these, ethyl decanoate and ethyl octanoate belong to the medium-chain fatty acid ethyl esters group with a low-odor threshold and low-volatility characteristics, which can effectively enhance the fruit aromas of fruit wines [54]. The synthesis of ethyl decanoate and ethyl octanoate is related to the metabolism of the fatty acid acyl and acetyl-CoA pathways, which are catalyzed by the ethanol O-acyltransferase EEB1 and O-acyltransferase EHT1 [6]. Phenylethyl alcohol (creating rose and honey notes) is one of the major higher alcohols in fruit wines, and it is synthesized via the Shikimate and Ehrlich pathways in *S. cerevisiae* cells [55]. The 11 °Brix Hu, 11 °Brix Kq, and 25 °C–20 °Brix Hu strawberry wines were all located in the first quadrant with isoamyl acetate, benzaldehyde, and texanol isobutyrate. These key flavor compounds could enhance banana, almond, and caramel notes in strawberry wines [31,56]. The 18 °C–20 °Brix Hu, 25 °C–20 °Brix Kq, and 18 °C–20 °Brix Kq strawberry wines were positioned in the fourth quadrant and had positive associations with linalool contents, nerol, nerolidol, D-citronellol, β-damascenone, 2,4-Di-tert-butylphenol, ethyl oleate, and γ-decalactone. The accumulation of these key flavor compounds was most likely due to the superior β-glucosidase-producing capacity of the Hu and Kq strains [10]. β-glucosidase catalyzes the hydrolysis of various glycosidic bonds, promoting the synthesis of free terpene alcohols and unique fatty esters during fruit wine fermentation [57]. In addition, the contents of linalool (549.96 ± 81.66 μg/L), nerol (379.10 ± 21.82 μg/L), and D-citronellol (105.87 ± 9.54 μg/L) were the highest in the 18 °C–20 °Brix Hu strawberry wines (Table S1), and these positively contributed to the enhancement of the sweetness and orange blossom and rose notes of the samples [58]. The 20 °Brix Kq groups were closer to β-damascenone and ethyl oleate, with β-damascenone (26.78–39.66 μg/L) being well above the detection threshold (0.002 μg/L), which could have significantly enhanced the rose notes of the strawberry wines [59]. The results demonstrated that the 20 °Brix strawberry wines contained more key flavor compounds, while the key aroma characteristics of the strawberry wine samples were significantly different. This was consistent with previous research findings where non-*Saccharomyces* yeast fermentation could alter the flavor profile and create flavor diversity in fruit wines [60]. It is worth noting that the application of the Hu and Kq strains in the fermentation of the strawberry wines remarkably enhanced the contents of the terpene alcohols and ketone, leading to an enhancement in the floral aroma characteristics of the strawberry wines.

#### 3.4.4. Redundancy Analysis

A redundancy analysis was used to identify the potential correlations between the VOCs and the physicochemical properties of the different strawberry wines (Figure 5d) [61]. The 18 °C–11 °Brix Sc and 20 °Brix Sc strawberry wines showed negative correlations with the titratable acid contents, which suggested that more titratable acid may have been converted to VOCs during the extended fermentation cycle (Figure 1) [13]. This was consistent with previous reports where different fermentation conditions had significant effects on the contents of VOCs in fermented fruit wines. In particular, the prolongation of the fermentation cycle contributed to the transformation and accumulation of VOCs in fermented fruit wines [62]. Moreover, there was a strong positive correlation between the 20 °Brix Sc strawberry wines and CO_2_ weight loss and ethanol contents. This result indicated that microbial species and fermentation conditions have significant effects on CO_2_ weight loss and ethanol contents in fermented fruit wines, and this was consistent with the previous findings reported by Jiang et al. [63] and Li et al. [64]. In addition, higher values of these two physicochemical properties indicated a more complete fermentation and promoted the formation of more ester compounds. Ester compounds are closely associated with the aromas of fruit wines and are one of the main factors influencing the formation of fruit wine flavor [65]. The contents of glycerol, glucose, reducing sugar, and SSC, as well as color intensity, were located in quadrant four, and they were positively correlated with the flavor profiles of the 11 °Brix Hu and 20 °Brix Kq strawberry wines. The correlations with color intensity and glycerol content could have been related to specific enzymes in the Hu and Kq strains. It has been previously demonstrated that non-*Saccharomyces* yeasts secrete specific enzymes during fermentation, including esterases, β-glucosidases, lipases, and proteases, which directly affect the production and accumulation of metabolites in fruit wines, thereby influencing the organoleptic quality and flavor profile of the fruit wine [66,67].

### 3.5. Electronic Nose Analysis

**Figure 6:**
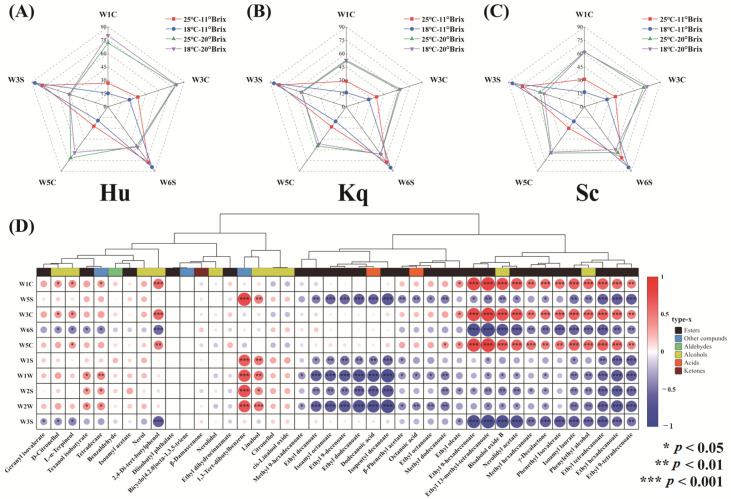
The E-nose analysis of the strawberry wine samples produced by the four different fermentation conditions: (**A**) distribution of the electronic nose sensor response values of the Hu strawberry wines, (**B**) distribution of the electronic nose sensor response values of the Kq strawberry wines, (**C**) distribution of the electronic nose sensor response values of the Sc strawberry wines, and (**D**) Spearman correlation analysis of the volatile organic compound contents and electronic nose sensor response values. The correlation coefficients are represented by colors, with red signifying a positive correlation and blue signifying a negative correlation.

An electronic nose was utilized to analyze the overall aromas of the strawberry wines, with the response values of the different sensors being related to the chemical compositions and the contents of the VOCs (Table S3) [61]. Compared with the 11 °Brix strawberry wines, the 20 °Brix strawberry wines had higher response values for the W1C, W3C, and W5C sensors (Figure 6A–C). The heat map in Figure 6D shows the correlation analysis performed on the response values of the sensors with the contents of the C7–C22 VOCs in the strawberry wines. The response values of W1C, W3C, and W5C were significantly and positively correlated with the contents of ethyl 9-hexadecenoate, ethyl 13-methyl-tetradecanoate, bisabolol oxide B, nerolidyl acetate, isoamyl laurate, phenylethyl alcohol, ethyl tetradecanoate, and ethyl hexadecenoate in the strawberry wines (*p* < 0.001). In addition, the 11 °Brix strawberry wines had higher W3S and W6S response values. Interestingly, the response values of the W3S and W6S sensors were negatively correlated with all VOC contents of the strawberry wines. These results demonstrate a strong correlation between the sensor responses and the contents of eight VOCs, accelerating the development of electronic nose technology for analyzing the aromatic properties of food products [68].

## 4. Conclusions

This study demonstrated that indigenous Hu and Kq strains and fermentation conditions markedly affect the ethanol content, glycerol content, color intensity, active substance content, antioxidant capacity, and flavor profile of strawberry wine. Interestingly, the flavor diversity in strawberry wine was positively impacted by the Hu and Kq strains. The Hu and Kq strawberry wines contained higher contents of terpene alcohols and β-Damascenone, which endowed the distinctive orange blossom and rose notes. Moreover, the 18 °C–20 °Brix strawberry wines had the highest glycerol contents, color intensities, antioxidant capacities, and total VOC contents, all of which contributed to enhancing the overall quality and flavor intensity of the strawberry wine. Additionally, the 18 °C–20 °Brix Kq strawberry wine had the highest contents of β-Damascenone, γ-Decalactone, ethyl hexadecanoate, and ethyl oleate (rOAV ≥ 1), endowing the strawberry wine with more peach, cream, and rose notes. Meanwhile, it also showed the highest DPPH and ABTS free-radical-scavenging capacities and the lowest ethanol content (6.42 ± 0.32%), which contributed to improving the quality of the low-alcohol strawberry wine.

The quality and flavor profile of strawberry wine could be significantly altered by adjusting the fermentation conditions, and here, we characterized that a fermentation temperature of 18 °C and strawberry juice with an initial SSC of 20 °Brix are the optimal conditions for fermenting strawberry wine. Moreover, the Kq strain demonstrated the ability to enhance the antioxidant capacity and flavor diversity of the strawberry wine while maintaining a low alcohol content, suggesting that it could be a viable alternative strain for the production of low-alcohol fruit wines. The Sc strawberry wines contained the highest total VOC contents under all fermentation conditions. Therefore, it is necessary to investigate the effect of the mixed fermentation process of Sc with the Kq and Hu strains, which could be a potential strategy for maintaining a high total VOC content while increasing the flavor diversity in fruit wine.

## Data Availability

The original contributions presented in this study are included in the article/Supplementary Materials. Further inquiries can be directed to the corresponding author.

## Supplementary Materials

The following supporting information can be downloaded at: https://www.mdpi.com/article/10.3390/foods14050886/s1, Table S1. Identification and relative contents of volatile organic compounds in strawberry wines with different fermentation trials. Table S2. Detailed information of the key flavor compounds with relative odor capacity value (rOAV) ≥ 1. Table S3. The description of E-nose sensitivity [69,70,71,72,73,74].

## References

[1] Bebek Markovinović A., Brčić Karačonji I., Jurica K., Lasić D., Skendrović Babojelić M., Duralija B., Šic Žlabur J., Putnik P., Bursać Kovačević D. (2022). Strawberry Tree Fruits and Leaves (*Arbutus unedo* L.) as Raw Material for Sustainable Functional Food Processing: A Review. Horticulturae.

[2] Qaderi R., Mezzetti B., Capocasa F., Mazzoni L. (2023). Stability of Strawberry Fruit (*Fragaria* x *ananassa* Duch.) Nutritional Quality at Different Storage Conditions. Appl. Sci..

[3] Bezerra M., Ribeiro M., Cosme F., Nunes F.M. (2024). Overview of the Distinctive Characteristics of Strawberry, Raspberry, and Blueberry in Berries, Berry Wines, and Berry Spirits. Compr. Rev. Food Sci. Food Saf..

[4] Bi P., Sun W., Li S., Liu X., Tian Y., Long F., Zhang Z., Guo J. (2024). Characterization of the Effect of Non-*Saccharomyces cerevisiaes* on the Non-Volatile Constituents and Volatile Profiles of Low-Alcoholic Pomegranate Beverages. Food Biosci..

[5] Klimczak K., Cioch-Skoneczny M., Ciosek A., Poreda A. (2024). Application of Non-*Saccharomyces* Yeast for the Production of Low-Alcohol Beer. Foods.

[6] Wang X., Fan G., Peng Y., Xu N., Xie Y., Zhou H., Liang H., Zhan J., Huang W., You Y. (2023). Mechanisms and Effects of Non-*Saccharomyces* Yeast Fermentation on the Aromatic Profile of Wine. J. Food Compos. Anal..

[7] Yang W., Liu S., Marsol-Vall A., Tähti R., Laaksonen O., Karhu S., Yang B., Ma X. (2021). Chemical Composition, Sensory Profile and Antioxidant Capacity of Low-Alcohol Strawberry Beverages Fermented with *Saccharomyces cerevisiae* and *Torulaspora delbrueckii*. LWT.

[8] Alonso-del-Real J., Lairon-Peris M., Barrio E., Querol A. (2017). Effect of temperature on the prevalence of *Saccharomyces* non *cerevisiae* species against a *S. cerevisiae* wine strain in wine fermentation: Competition, physiological fitness, and influence in final wine composition. Front. Microbiol..

[9] Gschaedler A., Iñiguez-Muñoz L.E., Flores-Flores N.Y., Kirchmayr M., Arellano-Plaza M. (2021). Use of Non-*Saccharomyces* Yeasts in Cider Fermentation: Importance of the Nutrients Addition to Obtain an Efficient Fermentation. Int. J. Food Microbiol..

[10] Yang B., Liu S., Zang H., Dai Y., Zhang S., Lin X., Liang H., Chen Y. (2024). Flavor Profile and Quality of Strawberry Wine Are Improved through Sequential Fermentation with Indigenous Non-*Saccharomyces* Yeasts and *Saccharomyces cerevisiae*. Food Biosci..

[11] Deed R.C., Fedrizzi B., Gardner R.C. (2017). Influence of Fermentation Temperature, Yeast Strain, and Grape Juice on the Aroma Chemistry and Sensory Profile of Sauvignon Blanc Wines. J. Agric. Food Chem..

[12] Wei J., Zhang Y., Wang Y., Ju H., Niu C., Song Z., Yuan Y., Yue T. (2020). Assessment of Chemical Composition and Sensorial Properties of Ciders Fermented with Different Non-*Saccharomyces* Yeasts in Pure and Mixed Fermentations. Int. J. Food Microbiol..

[13] Wu Y., Li Z., Zou S., Dong L., Lin X., Chen Y., Zhang S., Ji C., Liang H. (2023). Chemical Composition and Flavor Characteristics of Cider Fermented with *Saccharomyces cerevisiae* and Non-*Saccharomyces cerevisiae*. Foods.

[14] Yang X., Zhao F., Yang L., Li J., Zhu X. (2022). Enhancement of the Aroma in Low-Alcohol Apple-Blended Pear Wine Mixed Fermented with *Saccharomyces cerevisiae* and Non-*Saccharomyces* Yeasts. LWT.

[15] Qiu S., Chen K., Liu C., Wang Y., Chen T., Yan G., Li J. (2022). Non-*Saccharomyces* Yeasts Highly Contribute to Characterisation of Flavour Profiles in Greengage Fermentation. Food Res. Int..

[16] Sun X., Liu L., Zhao Y., Ma T., Zhao F., Huang W., Zhan J. (2016). Effect of Copper Stress on Growth Characteristics and Fermentation Properties of *Saccharomyces cerevisiae* and the Pathway of Copper Adsorption during Wine Fermentation. Food Chem..

[17] Jennings P.A., Mullen C.A., Roy M. (2010). Titration and Measurement. Encyclopedia of Life Sciences.

[18] Deshavath N.N., Mukherjee G., Goud V.V., Veeranki V.D., Sastri C.V. (2020). Pitfalls in the 3, 5-Dinitrosalicylic Acid (DNS) Assay for the Reducing Sugars: Interference of Furfural and 5-Hydroxymethylfurfural. Int. J. Biol. Macromol..

[19] Liu S., Laaksonen O., Kortesniemi M., Kalpio M., Yang B. (2018). Chemical Composition of Bilberry Wine Fermented with Non-*Saccharomyces* Yeasts (*Torulaspora delbrueckii* and *Schizosaccharomyces pombe*) and *Saccharomyces cerevisiae* in Pure, Sequential and Mixed Fermentations. Food Chem..

[20] Navajas-Porras B., Pérez-Burillo S., Morales-Pérez J., Rufián-Henares J.A., Pastoriza S. (2020). Relationship of Quality Parameters, Antioxidant Capacity and Total Phenolic Content of EVOO with Ripening State and Olive Variety. Food Chem..

[21] Wu Y., Xu L., Liu X., Hasan K.M.F., Li H., Zhou S., Zhang Q., Zhou Y. (2021). Effect of Thermosonication Treatment on Blueberry Juice Quality: Total Phenolics, Flavonoids, Anthocyanin, and Antioxidant capacity. LWT.

[22] Nogueira D.P., Jiménez-Moreno N., Esparza I., Moler J.A., Ferreira-Santos P., Sagües A., Teixeira J.A., Ancín-Azpilicueta C. (2023). Evaluation of Grape Stems and Grape Stem Extracts for Sulfur Dioxide Replacement during Grape Wine Production. Curr. Res. Food Sci..

[23] Zheng X., Chi H., Ma S., Zhao L., Cai S. (2023). Identification of Novel α-Glucosidase Inhibitory Peptides in Rice Wine and Their Antioxidant capacities Using in Silico and in Vitro Analyses. LWT.

[24] Sun Z., Cong Y., Li T., Meng X., Zhang F. (2022). Enhancement of Nutritional, Sensory and Storage Stability by Lactic Fermentation of Auricularia Auricula. J. Sci. Food Agric..

[25] Wang X.-C., Li A.-H., Dizy M., Ullah N., Sun W.-X., Tao Y.-S. (2017). Evaluation of Aroma Enhancement for “Ecolly” Dry White Wines by Mixed Inoculation of Selected Rhodotorula Mucilaginosa and *Saccharomyces cerevisiae*. Food Chem..

[26] Liu R., Liu Y., Zhu Y., Kortesniemi M., Zhu B., Li H. (2022). Aromatic Characteristics of Passion Fruit Wines Measured by E-Nose, GC-Quadrupole MS, GC-Orbitrap-MS and Sensory Evaluation. Foods.

[27] Jiang X., Lu Y., Liu S.Q. (2020). Effects of Pectinase Treatment on the Physicochemical and Oenological Properties of Red Dragon Fruit Wine Fermented with *Torulaspora delbrueckii*. LWT.

[28] Maksim Z., Matthias R. (2018). Cell size and morphological properties of yeast *Saccharomyces cerevisiae* in relation to growth temperature. FEMS Yeast Res..

[29] Sadras V.O., Petrie P.R., Moran M.A. (2013). Effects of Elevated Temperature in Grapevine. II Juice PH, Titratable Acidity and Wine Sensory Attributes. Aust. J. Grape Wine Res..

[30] Torija M. (2003). Effects of Fermentation Temperature on the Strain Population of *Saccharomyces cerevisiae*. Int. J. Food Microbiol..

[31] Han S., Yang J., Choi K., Kim J., Adhikari K., Lee J. (2022). Chemical Analysis of Commercial White Wines and Its Relationship with Consumer Acceptability. Foods.

[32] Du Q., Ye D., Zang X., Nan H., Liu Y. (2022). Effect of Low Temperature on the Shaping of Yeast-Derived Metabolite Compositions during Wine Fermentation. Food Res. Int..

[33] Clarke S., Bosman G., du Toit W., Aleixandre-Tudo J.L. (2023). White Wine Phenolics: Current Methods of Analysis. J. Sci. Food Agric..

[34] Bao Y., Zhang M., Chen W., Chen H., Chen W., Zhong Q. (2021). Screening and Evaluation of Suitable Non-*Saccharomyces* Yeast for Aroma Improvement of Fermented Mango Juice. Food Biosci..

[35] Sun W., Chen X., Feng S., Bi P., Han J., Li S., Liu X., Zhang Z., Long F., Guo J. (2024). Effect of Sequential Fermentation with Indigenous Non-*Saccharomyces cerevisiae* Combinations and *Saccharomyces cerevisiae* on the Chemical Composition and Aroma Compounds Evolution of Kiwifruit Wine. Food Chem..

[36] Massera A., Assof M., Sari S., Ciklic I., Mercado L., Jofré V., Combina M. (2021). Effect of Low Temperature Fermentation on the Yeast-Derived Volatile Aroma Composition and Sensory Profile in Merlot Wines. LWT.

[37] Samoticha J., Wojdyło A., Chmielewska J., Nofer J. (2019). Effect of Different Yeast Strains and Temperature of Fermentation on Basic Enological Parameters, Polyphenols and Volatile Compounds of Aurore White Wine. Foods.

[38] Zeinali S., Natalia Wieczorek M., Pawliszyn J. (2022). Free versus Droplet-Bound Aroma Compounds in Sparkling Beverages. Food Chem..

[39] Synos K., Reynolds A.G., Bowen A.J. (2015). Effect of Yeast Strain on Aroma Compounds in Cabernet Franc Icewines. LWT Food Sci. Technol..

[40] Feng Y., Liu M., Ouyang Y., Zhao X., Ju Y., Fang Y. (2015). Comparative Study of Aromatic Compounds in Fruit Wines from Raspberry, Strawberry, and Mulberry in Central Shaanxi Area. Food Nutr. Res..

[41] Aubert C., Bruaut M., Chalot G., Cottet V. (2021). Impact of maturity stage at harvest on the main physicochemical characteristics, the levels of vitamin C, polyphenols and volatiles and the sensory quality of Gariguette strawberry. Eur. Food Res. Technol..

[42] Cozzolino R., Pace B., Palumbo M., Laurino C., Picariello G., Siano F., De Giulio B., Pelosi S., Cefola M. (2021). Profiles of Volatile and Phenolic Compounds as Markers of Ripening Stage in Candonga Strawberries. Foods.

[43] Wu L., Wang X., Hao J., Zhu N., Wang M. (2023). Geographical Indication Characteristics of Aroma and Phenolic Acids of the Changping Strawberry. Foods.

[44] Neri F., Cappellin L., Spadoni A., Cameldi I., Algarra Alarcon A., Aprea E., Romano A., Gasperi F., Biasioli F. (2015). Role of Strawberry Volatile Organic Compounds in the Development of Botrytis Cinerea Infection. Plant Pathol..

[45] Prat L., Espinoza M.I., Agosin E., Silva H. (2014). Identification of Volatile Compounds Associated with the Aroma of White Strawberries (*Fragaria chiloensis*). J. Sci. Food Agric..

[46] Guo C., Deng H., Li E. (2024). Removal of Acetic Acid in Mulberry Wine by Co-Inoculating *Saccharomyces cerevisiae* with Indigenous Non-*Saccharomyces* Yeast. Food Biosci..

[47] Peng B., Li F., Cui L., Guo Y. (2015). Effects of Fermentation Temperature on Key Aroma Compounds and Sensory Properties of Apple Wine. J. Food Sci..

[48] Wang D., Chen L., Yang F., Wang H., Wang L. (2019). Yeasts and Their Importance to the Flavour of Traditional Chinese Liquor: A Review. J. Inst. Brew..

[49] Reboredo-Rodríguez P., González-Barreiro C., Rial-Otero R., Cancho-Grande B., Simal-Gándara J. (2015). Effects of Sugar Concentration Processes in Grapes and Wine Aging on Aroma Compounds of Sweet Wines—A Review. Crit. Rev. Food Sci. Nutr..

[50] Zhang B., Tang C., Yang D., Liu H., Xue J., Duan C., Yan G. (2022). Effects of Three Indigenous Non-*Saccharomyces* Yeasts and Their Pairwise Combinations in Co-Fermentation with *Saccharomyces cerevisiae* on Volatile Compounds of Petit Manseng Wines. Food Chem..

[51] Tian M., Lin K., Yang L., Jiang B., Zhang B., Zhu X., Ren D., Yu H. (2023). Characterization of Key Aroma Compounds in Gray Sufu Fermented Using Leuconostoc Mesenteroides Subsp. Mesenteroides F24 as a Starter Culture. Food Chem. X.

[52] Wang Y., He Y., Liu Y., Wang D. (2022). Analyzing Volatile Compounds of Young and Mature Docynia Delavayi Fruit by HS-SPME-GC-MS and ROAV. Foods.

[53] Zhao Z., Hao Y., Liu Y., Shi Y., Lin X., Wang L., Wen P., Hu X., Li J. (2023). Comprehensive Evaluation of Aroma and Taste Properties of Different Parts from the Wampee Fruit. Food Chem. X.

[54] Xue S.-J., Zhang J.-R., Zhang R.-X., Qin Y., Yang X.-B., Jin G.-J., Tao Y.-S. (2022). Oxidation-Reduction Potential Affects Medium-Chain Fatty Acid Ethyl Ester Production during Wine Alcohol Fermentation. Food Res. Int..

[55] Dai J., Li K., Song N., Yao W., Xia H., Yang Q., Zhang X., Li X., Wang Z., Yao L. (2020). *Zygosaccharomyces rouxii*, an Aromatic Yeast Isolated from Chili Sauce, Is Able to Biosynthesize 2-Phenylethanol via the Shikimate or Ehrlich Pathways. Front. Microbiol..

[56] Tian T., Sun J., Wu D., Xiao J., Lu J. (2021). Objective Measures of Greengage Wine Quality: From Taste-Active Compound and Aroma-Active Compound to Sensory Profiles. Food Chem..

[57] Zhang P., Zhang R., Sirisena S., Gan R., Fang Z. (2021). Beta-Glucosidase capacity of Wine Yeasts and Its Impacts on Wine Volatiles and Phenolics: A Mini-Review. Food Microbiol..

[58] Huang M., Liu X., Li X., Sheng X., Li T., Tang W., Yu Z., Wang Y. (2022). Effect of *Hanseniaspora uvarum*–*Saccharomyces cerevisiae* Mixed Fermentation on Aroma Characteristics of Rosa Roxburghii Tratt, Blueberry, and Plum Wines. Molecules.

[59] Chaumont-Olive P., Sánchez-Quesada J., Collado Pérez A.M., Cossy J. (2021). Synthetic Approaches to the Damascone and Damascenone Isomers. Tetrahedron.

[60] Cândido da Silva M.C., de Brito Araújo Carvalho A.J., Cardoso Viana A., Almeida dos Anjos V.H., Prudêncio Dutra M.d.C., Pimentel T.C., Magnani M., dos Santos Lima M. (2024). Effects of Unconventional Non-*Saccharomyces* Yeast Fermentation on the Chemical Profile and Bioaccessibility of Watermelon Wine. Food Biosci..

[61] Cai W., Tang F., Guo Z., Guo X., Zhang Q., Zhao X., Ning M., Shan C. (2020). Effects of Pretreatment Methods and Leaching Methods on Jujube Wine Quality Detected by Electronic Senses and HS-SPME–GC–MS. Food Chem..

[62] Liu S., Lou Y., Li Y., Zhao Y., Feng X., Capozzi V., Laaksonen O., Yang B., Li P., Gu Q. (2023). Comparison of Anthocyanin and Volatile Organic Compounds in Juices and Fruit Wines Made from Blood Oranges (*Citrus sinensis* L. Osbeck) at Different Maturity Stages. Food Biosci..

[63] Jiang J., Yin R., Xie Y., Ma X., Cui M., Chen Y., Li Y., Hu Y., Niu J., Cheng W. (2024). Effects of Cofermentation of *Saccharomyces cerevisiae* and Different Lactic Acid Bacteria on the Organic Acid Content, Soluble Sugar Content, Biogenic Amines, Phenol Content, Antioxidant capacity and Aroma of Prune Wine. Food Chem. X.

[64] Li W., Guo Q., Zhao Y., Yue T., Yuan Y. (2024). Untargeted Metabolomics Combined with Chemometrics Reveals Distinct Metabolic Profiles across Two Sparkling Cider Fermentation Stages. Food Res. Int..

[65] Wang M., Wang C., Yang C., Peng L., Xie Q., Zheng R., Dai Y., Liu S., Peng X. (2021). Effects of Lactobacillus Plantarum C7 and Staphylococcus Warneri S6 on Flavor Quality and Bacterial Diversity of Fermented Meat Rice, a Traditional Chinese Food. Food Res. Int..

[66] García M., Esteve-Zarzoso B., Arroyo T. (2016). Non-Saccharomyces Yeasts: Biotechnological Role for Wine Production. Grape and Wine Biotechnology.

[67] Ge X., Liu Y., Wang X., Gao C., Mu J., Wang W., Wang J. (2024). Correlations between Microbes with Volatile Compounds and Physicochemical Indicators of Cabernet Sauvignon Wines Fermented with Different Starters. LWT.

[68] Wang J., Wei B., Zhai Y., Li K., Wang C. (2024). Non-volatile and Volatile Compound Changes in Blueberry Juice Inoculated with Different Lactic Acid Bacteria Strains. J. Sci. Food Agric..

[69] Van Gemert L.J. (2011). Odour thresholds: Compilations of Odour Threshold Values in Air, Water and Other Media.

[70] Li S., Bi P., Sun N., Gao Z., Chen X., Guo J. (2022). Effect of sequential fermentation with four non-*Saccharomyces* and *Saccharomyces cerevisiae* on nutritional characteristics and flavor profiles of kiwi wines. J. Food Compos. Anal..

[71] Li S., Chen X., Gao Z., Zhang Z., Bi P., Guo J. (2023). Enhancing antioxidant activity and fragrant profile of low-ethanol kiwi wine via sequential culture of indigenous *Zygosaccharomyces rouxii* and *Saccharomyces cerevisiae*. Food Biosci..

[72] Wang X., Chen J., Ge X., Fu X., Dang C., Wang J., Liu Y. (2023). Sequential fermentation with indigenous non-*Saccharomyces* yeasts and *Saccharomyces cerevisiae* for flavor and quality enhancement of Longyan dry white wine. Food Biosci..

[73] http://www.flavornet.org/flavornet.html.

[74] https://china.guidechem.com/dict/.

