# Supplementary material for: The Antioxidant Capacity and Flavor Diversity of Strawberry Wine Are Improved Through Fermentation with the Indigenous Non-Saccharomyces Yeasts Hanseniaspora uvarum and Kurtzmaniella quercitrusa
Source: Foods. 2025 Mar 5;14(5):886. doi: 10.3390/foods14050886 (PMC11899187; doi:10.3390/foods14050886)
Supplement: Supplementary file 1 [file foods-14-00886-s001.zip › foods-3495849-supplementary.pdf]

**Supplementary Information**

Table S1 Identification and relative contents of volatile organic compounds in strawberry wines with different fermentation trials.

| Compounds | Relative content (μg/L) | | | | Relative content (μg/L) | | | | Relative content (μg/L) | | | |
|---|---|---|---|---|---|---|---|---|---|---|---|---|
| | Hu | | | | Kq | | | | Sc | | | |
| | 25 ºC − 11 ºBrix | 18 ºC − 11 ºBrix | 25 ºC − 20 ºBrix | 18 ºC–20 ºBrix | 25 ºC − 11 ºBrix | 18 ºC − 11 ºBrix | 25 ºC–20 ºBrix | 18 ºC–20 ºBrix | 25 ºC − 11 ºBrix | 18 ºC − 11 ºBrix | 25 ºC–20 ºBrix | 18 ºC–20 ºBrix |
| **Aldehydes** | | | | | | | | | | | | |
| Benzaldehyde | 381.3±661.4 | 173.6±102.7 | 87.0±6.0 | 232.3±103.6 | 70.8±25.5 | 17.3±3.3 | 54.2±1.8 | 120.2±13.8 | 162.4±7.0 | 73.7±30.4 | 193.5±36.4 | 41.3±3.8 |
| **Ketones** | | | | | | | | | | | | |
| β-Damascenone | ND | ND | ND | ND | 5.0±0.5 | 9.1±3.5 | 26.8±6.4 | 39.7±5.1 | ND | ND | ND | ND |
| **Acids** | | | | | | | | | | | | |
| Octanoic acid | 128.1±37.9 | 18.5±0.4 | 102.9±25.3 | 99.9±77.7 | ND | ND | ND | ND | 1865.2±254.6 | 1569.1±227.8 | 1388.2±149.6 | 2911.3±22.4 |
| Dodecanoic acid | 131.9±27.9 | ND | ND | ND | 18.2±19.0 | 15.2±10.3 | 112.1±28.0 | 114.1±2.1 | 131.4±48.6 | 248.8±54.5 | 218.8±71.4 | 528.3±240.4 |
| **Alcohols** | | | | | | | | | | | | |
| cis-Linalool oxide | 8.8±0.2 | ND | ND | ND | ND | ND | ND | ND | ND | ND | ND | ND |
| Linalool | 306.4±55.9 | 239.0±99.0 | 189.3±57.1 | 550.0±81.7 | 174.8±59.8 | 327.7±60.6 | 238.4±108.2 | 338.8±6.6 | 104.3±2.4 | 125.7±4.4 | 88.6±29.4 | 230.4±53.7 |
| Phenylethyl alcohol | 770.2±196.6 | 374.6±93.3 | 1511.2±75.6 | 3440.5±712.8 | 1432.9±415.1 | 822.8±82.5 | 1752.3±287.9 | 1715.0±68.6 | 5582.6±669.9 | 1854.5±214.1 | 3987.4±270.1 | 5012.9±1663.7 |
| L-α-Terpineol | 65.3±1.6 | 43.0±2.4 | 123.6±51.0 | 142.3±7.8 | 87.2±6.7 | 47.4±13.9 | 176.6±17.6 | 179.2±6.5 | 29.9±4.0 | 25.2±3.8 | 21.7±13.3 | 97.7±49.8 |

| | | | | | | | | | | | | |
|---|---|---|---|---|---|---|---|---|---|---|---|---|
| Citronellol | 23.9±2.2 | ND | ND | ND | ND | ND | ND | ND | ND | ND | ND | ND |
| D-Citronellol | 20.9±50.9 | 5.2±1.3 | 113.5±73.4 | 105.9±9.5 | ND | 7.5±0.8 | 83.9±42.3 | ND | ND | ND | ND | ND |
| Nerol | ND | ND | ND | 379.1±21.8 | ND | 12.3±2.8 | 84.0±36.5 | 43.9±13.3 | ND | ND | ND | ND |
| Nerolidol | 163.3±18.7 | 107.2±3.8 | 48.6±12.1 | 155.2±27.9 | 67.6±11.2 | 72.0±12.4 | 140.4±33.6 | 127.4±14.0 | 86.0±11.9 | 167.8±37.3 | 78.7±9.3 | 130.2±49.7 |
| Bisabolol oxide B | 66.2±20.0 | 33.7±14.5 | 110.0±28.2 | 147.3±53.3 | 36.2±7.5 | 31.8±5.8 | 204.7±44.0 | 266.4±37.5 | 58.4±11.9 | 49.5±9.0 | 71.0±21.3 | 109.6±41.4 |
| **Esters** | | | | | | | | | | | | |
| Isoamyl acetate | 157.6±64.9 | 146.5±66.3 | 69.6±7.3 | 128.9±79.4 | 18.8±19.4 | ND | ND | ND | 15.7±4.2 | 60.7±10.0 | 61.1±24.2 | 75.6±12.8 |
| Ethyl octanoate | 122.8±35.6 | 20.3±0.8 | 116.0±52.1 | 168.0±28.3 | ND | ND | ND | ND | 806.5±305.0 | 1253.6±437.1 | 1072.4±143.0 | 1776.6±235.8 |
| β-Phenethyl acetate | 453.0±218.3 | 197.0±74.0 | 656.9±265.2 | 28.3±3.1 | 91.9±37.8 | ND | ND | ND | 531.4±56.4 | 516.2±117.7 | 1175.2±66.7 | 943.4±245.1 |
| Phenethyl Isovalerate | 60.8±8.2 | 2.2±0.5 | 122.7±62.0 | 81.2±21.5 | ND | ND | 43.1±22.7 | 33.2±1.3 | 69.7±18.4 | 17.3±1.1 | ND | 134.2±25.4 |
| Ethyl dihydrocinnamate | ND | ND | ND | ND | ND | 3.6±0.9 | ND | ND | ND | ND | 16.4±4.4 | 9.2±3.8 |
| Ethyl 9-decenoate | ND | ND | ND | ND | ND | ND | ND | ND | 1385.0±309.2 | 4332.5±1688.2 | 2537.4±164.2 | 2888.6±1506.7 |
| Ethyl decanoate | 231.2±104.5 | ND | ND | ND | 662.2±108.8 | 23.3±8.9 | 83.6±33.0 | 76.9±23.5 | 1208.9±381.7 | 2998.2±1165.6 | 1267.6±43.6 | 1967.7±54.1 |
| Isoamyl octanoate | ND | ND | ND | ND | ND | ND | ND | ND | 58.2±18.1 | 60.2±34.6 | 34.8±11.1 | 57.4±3.8 |
| γ-Decalactone | 951.0±177.5 | 608.2±121.2 | 1007.1±239.2 | 1591.4±329.1 | 501.7±85.9 | 624.3±104.8 | 1431.9±147.2 | 1650.3±232.9 | 815.9±169.0 | 1056.4±174.6 | 864.6±131.4 | 1084.9±447.4 |

| | | | | | | | | | | | | |
|---|---|---|---|---|---|---|---|---|---|---|---|---|
| Nerolidyl acetate | 34.9±0.4 | 13.8±0.4 | 34.5±9.8 | 61.0±23.7 | 15.1±1.2 | 15.7±5.3 | 72.2±26.9 | 53.8±4.5 | 38.0±8.3 | 21.6±2.8 | 28.2±6.5 | 54.5±7.8 |
| Methyl dodecanoate | ND | ND | ND | ND | ND | ND | ND | ND | 24.3±4.8 | ND | 11.7±2.0 | 30.1±8.1 |
| Ethyl dodecanoate | 474.4±93.8 | ND | ND | ND | 246.9±42.3 | 48.9±13.9 | 371.4±53.4 | 509.0±45.1 | 905.6±161.5 | 2822.7±940.9 | 2921.1±641.9 | 4654.0±1032.7 |
| Texanol isobutyrate | ND | 246.2±121.4 | 310.3±114.4 | 300.1±89.7 | ND | ND | ND | ND | ND | ND | ND | ND |
| Isopentyl decanoate | ND | ND | ND | ND | ND | 1.7±0.6 | 42.9±21.7 | 47.3±11.3 | 84.8±23.6 | 258.3±75.1 | 265.1±56.5 | 373.4±63.1 |
| Geranyl isovalerate | 90.9±16.0 | 39.6±10.3 | 85.1±36.3 | 173.6±41.5 | 40.8±9.9 | 20.2±4.3 | 186.3±44.8 | 203.0±29.2 | 73.3±21.7 | ND | ND | ND |
| Ethyl 9-tetradecenoate | 44.0±5.1 | 26.1±6.2 | 116.7±0.0 | 193.8±67.2 | 3.2±0.7 | 3.0±0.9 | 240.2±98.3 | 288.0±91.6 | 33.5±6.6 | 335.6±142.0 | 226.1±70.0 | 203.6±44.2 |
| Ethyl tetradecanoate | 146.6±46.9 | 80.4±28.9 | 367.0±325.5 | 467.8±290.4 | 69.8±9.8 | 68.6±11.3 | 602.4±162.3 | 967.2±288.0 | 126.7±24.0 | 266.1±64.9 | 1296.7±347.0 | 1092.7±143.0 |
| Isoamyl laurate | 19.2±7.6 | 7.7±2.7 | 72.6±29.7 | 98.0±23.7 | 7.6±4.6 | 44.9±58.1 | 69.9±15.1 | 126.8±21.1 | 28.3±6.5 | 90.5±22.3 | 344.5±150.2 | 418.3±72.3 |
| Diisobutyl phthalate | ND | ND | ND | ND | ND | ND | ND | ND | ND | ND | ND | ND |
| Ethyl 13-methyl-tetradecanoate | 35.8±8.9 | 11.2±3.9 | 209.7±140.3 | 252.9±155.8 | 17.3±1.3 | 3.5±1.5 | 210.6±58.2 | 392.6±133.6 | 17.5±5.5 | 12.4±4.2 | 698.1±168.3 | 453.2±95.6 |
| Methyl 9-hexadecanoate | 11.0±1.6 | ND | ND | ND | ND | ND | ND | ND | 9.7±2.1 | 6.1±0.4 | ND | 121.6±48.6 |
| Methyl hexadecanoate | 15.8±2.8 | 2.9±1.4 | 49.4±0.0 | 52.7±13.4 | 12.5±4.9 | 123.7±89.7 | 93.2±18.3 | 160.8±32.3 | 17.9±5.6 | 23.1±9.0 | 182.4±55.1 | 68.6±22.6 |
| Ethyl 9-hexadecenoate | 436.9±55.4 | 158.2±75.1 | 796.5±501.3 | 1028.3±182.7 | ND | 260.8±83.8 | 725.0±150.2 | 840.0±231.8 | 411.8±99.8 | 15.6±6.6 | 2455.3±337.3 | 1255.2±355.4 |

| | | | | | | | | | | | | |
|---|---|---|---|---|---|---|---|---|---|---|---|---|
| Ethyl hexadecanoate | 446.2±104.5 | 158.9±64.4 | 791.5±484.1 | 1330.7±46.1 | 363.8±79.3 | 89.2±113.0 | 2311.4±481.6 | 2195.6±282.7 | 516.8±112.2 | 1670.6±577.9 | 2844.9±749.8 | 1976.3±644.9 |
| Ethyl oleate | 77.5±2.6 | 54.5±31.5 | 128.2±54.4 | 194.0±47.3 | 142.7±21.9 | 250.5±58.7 | 2087.7±288.5 | 1791.2±24.6 | 90.3±25.8 | 156.1±44.9 | 392.2±121.7 | 255.0±77.7 |
| **Other compounds** | | | | | | | | | | | | |
| 2,4-Di-tert-butylphenol | 131.4±15.0 | 52.6±19.7 | 153.9±11.9 | 245.3±55.7 | 88.4±20.5 | 71.0±30.2 | 212.0±26.3 | 305.2±83.6 | 197.3±21.9 | 44.1±7.0 | 47.0±7.4 | 103.4±25.2 |
| Bicyclo [4.2.0] octa-1,3,5-triene | ND | ND | ND | ND | ND | ND | ND | ND | ND | ND | ND | ND |
| 1,3-Tert-dibutylbenzene | 14.5±3.3 | 6.0±0.8 | ND | 64.0±4.3 | 16.8±8.2 | ND | ND | ND | ND | ND | ND | ND |
| Tetradecane | ND | 45.5±19.5 | 187.6±55.4 | 243.5±97.7 | ND | ND | ND | ND | ND | ND | ND | ND |
| **All compounds** | 6021.9±1063.6$^{ab}$ | 2872.8±439.0$^{a}$ | 7561.4±2359.5$^{b}$ | 11955.8±2134.4$^{b}$ | 4192.5±599.1$^{a}$ | 3016.0±288.9$^{a}$ | 11657.2±1923.7$^{b}$ | 12650.6±751.4$^{b}$ | 15387.5±2734.6$^{a}$ | 20132.3±5870.6$^{ab}$ | 24790.7±2803.5$^{ab}$ | 29059.4±5812.1$^{b}$ |

The values shown represent the averages of triplicate samples (mean ± SD).

The values with different superscript roman letters in the same row are significantly different according to the Duncan test ($p < 0.05$).

ND means the compound was not detected by GC-MS.

Table S2 Detailed information of the key flavor compounds with relative odor capacity value (rOAV) ≥ 1

| Compounds | CAS | RI | Threshold (μg/L)[A] | Odor Description |
|---|---|---|---|---|
| **Aldehydes** | | | | |
| Benzaldehyde | 100-52-7 | 962 | 300 | Almond, burnt sugar[B] |
| **Ketones** | | | | |
| β-Damascenone | 23726-93-4 | 1385 | 0.002 | Honey apple, rose[E] |
| **Acids** | | | | |
| Octanoic acid | 124-07-2 | 1180 | 910.00 | Sweat[G] |
| **Alcohols** | | | | |
| Phenylethyl alcohol | 60-12-8 | 1116 | 140 | Citrus, rose, grassy[D] |
| Linalool | 78-70-6 | 1099 | 6 | Floral[B] |
| D-Citronellol | 1117-61-9 | 1197 | 40 | Rose[G] |
| Nerol | 106-25-2 | 1213 | 290 | Sweet[F] |
| Nerolidol | 7212-44-4 | 1570 | 10 | Rose, apple[G] |
| **Esters** | | | | |
| Isoamyl acetate | 123-92-2 | 876 | 2 | Banana[D] |
| Ethyl octanoate | 106-32-1 | 1173 | 19 | Fruity, fatty[F] |
| β-Phenethyl acetate | 103-45-7 | 1258 | 19 | Rose, honey, tobacco[F] |
| Ethyl decanoate | 110-38-3 | 1388 | 5 | Fruity, fatty, wax [E] |
| γ-Decalactone | 706-14-9 | 1470 | 1.1 | Peach[G] |
| Methyl dodecanoate | 111-82-0 | 1513 | 1.50 | Wine, floral[G] |
| Ethyl dodecanoate | 106-33-2 | 1595 | 400 | Pear, wine[C] |
| Texanol isobutyrate | 6846-50-0 | 1598 | 14 | NF |
| Ethyl tetradecanoate | 124-06-1 | 1786 | 180 | Fat, coconut[G] |

| | | | | |
|---|---|---|---|---|
| Ethyl hexadecanoate | 628-97-7 | 1985 | 2000 | Wax, cream[G] |
| Ethyl oleate | 111-62-6 | 2176 | 870 | Floral[G] |
| **Other compounds** | | | | |
| 2,4-Di-tert-butylphenol | 96-76-4 | 1514 | 500 | NF |

NF means that the relevant information was not found.

Odor threshold and odor description were obtained from literature and website: [A] (Van Gemert et al., 2011); [B] (Tian et al., 2021); [C] (Li et al., 2022); [D] (Li et al., 2023); [E] (Wang et al., 2023); [F] http://www.flavornet.org/flavornet.html (accessed August 17, 2024); [G] https://china.guidechem.com/dict/ (accessed August 17, 2024).

Table S3 The description of E-nose sensitivity

| Sensor | Description | Corresponding group threshold (mL • $m^{-3}$) |
|---|---|---|
| W1C | Sensitivity to aromatic ingredients, benzene | 10（$C_7H_8$） |
| W5S | Sensitive to nitrogen oxides | 1（$NO_2$） |
| W3C | Sensitive to ammonia, aromatic ingredients | 10（$C_6H_6$） |
| W6S | Selective mainly for hydrides | 100（$H_2$） |
| W5C | Sensitive to short-chain alkane aromatics | 1（$C_3H_8$） |
| W1S | Sensitive to methyl groups | 100（$CH_4$） |
| W1W | Sensitive to inorganic sulfides | 1（$H_2S$） |
| W2S | Sensitive to alcohols, aldehydes and ketones | 100（CO） |
| W2W | Sensitive to organic sulfides | 1（$H_2S$） |
| W3S | Sensitive to long chain alkanes | 10（$CH_4$） |

## References

Li, S., Bi, P., Sun, N., Gao, Z., Chen, X., & Guo, J. (2022). Effect of sequential fermentation with four non-*Saccharomyces* and *Saccharomyces cerevisiae* on nutritional characteristics and flavor profiles of kiwi wines. *Journal of Food Composition and Analysis*, *109*, 104480. https://doi.org/10.1016/j.jfca.2022.104480

Li, S., Chen, X., Gao, Z., Zhang, Z., Bi, P., & Guo, J. (2023). Enhancing antioxidant activity and fragrant profile of low-ethanol kiwi wine via sequential culture of indigenous *Zygosaccharomyces rouxii* and *Saccharomyces cerevisiae*. *Food Bioscience*, *51*, 102210. https://doi.org/10.1016/j.fbio.2022.102210

Tian, T., Sun, J., Wu, D., Xiao, J., & Lu, J. (2021). Objective measures of greengage wine quality: From taste-active compound and aroma-active compound to sensory profiles. *Food Chemistry*, *340*, 128179. https://doi.org/10.1016/j.foodchem.2020.128179

Van Gemert, L. J. (2011). Odour thresholds: *Compilations of odour threshold values in air, water and other media*, *2*. Oliemans Punter. ISBN: 979-90-810894-0-1

Wang, X., Chen, J., Ge, X., Fu, X., Dang, C., Wang, J., & Liu, Y. (2023). Sequential fermentation with indigenous non-*Saccharomyces* yeasts and *Saccharomyces cerevisiae* for flavor and quality enhancement of Longyan dry white wine. *Food Bioscience*, *55*, 102952.

https://doi.org/10.1016/j.fbio.2023.102952
